# The Role of Traditional Chinese Medicine in Arthritis Management: Why We Need Public Health and Health Services Research

**DOI:** 10.3389/fpubh.2020.597917

**Published:** 2020-12-17

**Authors:** Lu Yang, Jon Adams

**Affiliations:** ^1^School of Sociology and Population Sciences, University of Posts and Telecommunications, Nanjing, China; ^2^Faculty of Health, School of Public Health, Australian Research Centre in Complementary and Integrative Medicine (ARCCIM), University of Technology Sydney, Ultimo, NSW, Australia

**Keywords:** traditional Chinese medicine, arthritis, health services research, public health, care management

## Key Points

- There is limited evidence regarding the efficacy of TCM for arthritis, yet TCM such as Chinese herbal medicine and acupuncture is popular among arthritis patients
- Majority of TCM utilization by arthritis patients is concurrent to conventional medications and this raises the potential of a number of direct and indirect risks
- Direct and indirect risks of TCM use within wider arthritis patient care may be exacerbated by challenges around communication and disclosure
- We need a more sophisticated level of detail about how, when and why people use TCM for arthritis and further empirical work on this topic is required to help inform conventional health practitioners, policymakers, and patients to ensure safe, effective coordinated care for those with arthritis.

Arthritis has contributed significantly to the global disability burden associated with the musculoskeletal system (1). Comprised over 100 different diseases and conditions that affect joints, the surrounding and other connective tissues, arthritis is a leading cause of pain, functional disability and health services utilization in many countries (2). Moreover, arthritis is associated with reduced workplace productivity in higher-income countries, as well as in lower- and middle-income countries, causing subsequently worsening poverty (1). Furthermore, osteoarthritis (OA) and rheumatoid arthritis (RA) are the two most common forms of arthritis, exposing to a higher risk of comorbidities and mental problems, compared to the general population (3). Under these circumstances, challenges increase in the management of arthritis for patients with arthritis, healthcare providers and policy makers.

Traditional Chinese medicine (TCM), a comprehensive medical health system including acupuncture, Chinese herbal medicine, Chinese therapeutic massage, Tai Chi, Qi Gong, and a range of other health modalities has attracted increasing attention from arthritis sufferers and healthcare providers (4). However, there is limited evidence supporting the efficacy of TCM treatments for arthritis (5–7). While those studies appealing to a restricted evidence-base (limited to efficacy and pursued *via* randomized controlled trials and other experimental designs) may dismiss complementary medicine and TCM as irrelevant to arthritis care and investigation, a significant number of people with arthritis do use TCM in addition to their conventional medical treatments. Healthcare providers and patients with arthritis need to be aware of the potential direct and indirect risks associated with the concurrent use of multiple medications and practices. Indeed, the dismissal of complementary medicine and TCM denies a number of important practice realities and may well prove detrimental to the safety and quality of care provided to many with arthritis.

TCM has been developed since ancient China and has been incorporated into the western culture. In countries such as Australia, TCM has been recognized and registered under Australian Practitioner Health Regulation Agency (APHRA) and TCM has been incorporated into the 11th Revision of International Statistical Classification of Diseases and Related Health Problems (ICD-11) at the 72nd World Health Assembly held in Geneva, Switzerland in 2019 (8). This consideration and engagement partly reflects the reality of health care use in healthcare systems among WHO member states (9). Despite the acknowledged lack of evidence regarding efficacy for TCM use for arthritis, coping with arthritis remains a leading reason why US adults seek a range of complementary medicine treatments including TCM (2). Indeed, the popularity of TCM among those with arthritis has grown of late and the treatment of arthritis is the leading clinical reason why patients consult acupuncturists (10).

Substantial use of TCM among those with arthritis is reason enough to insist this topic be a core focus for public health and health services research, yet there are other circumstances that further spotlight the significance of investigating TCM use for arthritis, ideally *via* a multi-disciplinary perspective and approach. The majority of TCM use among those with arthritis occurs concurrently to conventional medical treatment (2)—a situation that introduces potential direct and indirect risks (11). There are direct risks related with TCM use for arthritis—regarding adverse events (e.g., itching, skin rashes, redness) from TCM patches, as well as TCM–conventional drug reactions (e.g., lassitude, nocturia, cold extremities) and indirect risks associated with the quality of TCM herbs and formulations owing to geographical or seasonal variations, as well as potential information about practitioner variability in TCM knowledge and communication between patients and practitioners (11).

Such risks are exacerbated by communication inefficiencies, both between patients and practitioners and across practitioners who may be engaged in patient care. While co-ordinated care between healthcare providers and patients with arthritis is often an ideal, research shows complementary medicine users tend not to disclose to their conventional health providers (2, 12) and conventional doctors tend not to enquire with patients regarding possible treatments they may be utilizing beyond the mainstream (13). A recent systematic review and meta-analysis focusing on disclosure of TCM use and other CAM use to medical providers indicated a 33% disclosure rate (12). Such findings appear to hold among those with arthritis and among those who provide their conventional medical care with regards to TCM (2).

The percentage of patients with arthritis using TCM ranges from 7.7 to 27.3% (2, 14). In light of these circumstances, it is imperative we know much more around how, when and why TCM is being sought, used and communicated within arthritis care. Indeed, further exploration of how TCM use and practice play out in daily routine health-seeking and care for those with arthritis is of benefit to all receiving and providing treatments, whether TCM or conventional in nature. It is essential for healthcare providers, particularly conventional doctors, to be fully informed of all patient's health-seeking information in order to avoid potential risks and inefficiencies and ensure optimal clinical care.

In response, there is an urgent need to broaden the research gaze on this topic—to move beyond a preoccupation with efficacy and trial design and to also embrace public health and health services research thinking and practice (15, 16). Drawing upon a range of established disciplines such as health geography, health psychology, epidemiology, and health sociology as well as a vast range of associated, rigorous methods of data collection and analyses it is possible to supplement the tight clinical focus of emerging research on TCM in arthritis to good effect. In a health research age where translation and implementation are rightfully occupying much attention and scholarship (17), a focus upon broadening perspectives and methodology should be welcomed by all. Moreover, the ability to move ever closer to positively changing the thinking and actions of stakeholders with a view to better health outcomes is itself only fully realized by such a multidisciplinary (perhaps truly transdisciplinary) research approach.

While not wanting to prescribe specific research designs and questions at the expense of others, a range of pertinent issues around TCM use in arthritis can be identified that require our urgent attention. We still know relatively little about the drivers and barriers to better communication and disclosure between arthritis patients and their clinicians regarding possible and/or active TCM use. Similarly, the communication and coordination of arthritis care across the conventional and complementary medical practitioner divide remains uncharted territory and, at a basic level, we still know little about the possible acceptance and/or success of implementation strategies and interventions with a view to improving practices and communication around this topic. While in the immediate term the choice of which specific areas to address may be open to debate, what is beyond question is that TCM use among those with arthritis is substantial, associated with a range of possible direct and indirect risks to patient care and as a consequence requires a broad multidisciplinary health research approach. The challenge for all providing care is to accommodate and embrace rigorous, scientific enquiry on this topic and to be open to at least discussing TCM use with their patients, regardless of whether they personally support or oppose TCM use. The bottom line is clear—a significant number of patients are already users of TCM for their arthritis and only through investigation and better understanding of the behaviors, motivations and outcome of such treatments and practices will we be able to move toward ensuring safe, effective co-ordinated care for those with arthritis.

## Author Contributions

LY: wrote the draft and the final manuscript. JA: read and criticized the draft. All authors read and approved the final manuscript.

## Conflict of Interest

The authors declare that the research was conducted in the absence of any commercial or financial relationships that could be construed as a potential conflict of interest.
